# Hypodermic Medication

**Published:** 1869-04

**Authors:** Edward Warren


### SELECTED ARTICLES.

### ARTICLE X.

### *Hypodermic Medication.*

Extreme views are entertained by the profession in regard to the subject of Hypodermic Medication. Its opponents and its advocates are equally enthusiastic in the maintenance of the most antagonistic opinions of its efficacy and availability. In the estimation of the *former* it is not simply one of the fashionable novelties of the day, smacking more of empiricism than of legitimate medicine, but a remedy of the most dangerous and unreliable character. The *latter* are almost fanatical in their advocacy of its utility, and of its claims to a conspicuous position among the grandest triumphs by which the history of our science has been illustrated. Between these two antagonistic factions there seems to be no possibility of a compromise—no neutral ground within which to bury their prejudices and heart-burnings. While reprobated by one as a *curse* it is regarded by the other as an inestimable *blessing* to the human family, and *neither* is at all disposed to recede from its extreme position.

As the natural result of these conflicting ideas, hypodermic injections are either too little or too much employed in the treatment of disease. Many physicians refuse, under any circumstances, to resort to them; while others use them indiscriminately in every case which falls to their care. The truth in this matter, as in many others, lies between the two extremes. There is nothing "dangerous or uncertain" about this method of medication, if it be managed judiciously. There is no justice in sneering at "novelties," whether they be fashionable or otherwise, simply because they conflict with long received opinions and deep-rooted prejudices. Every discovery in medicine is entitled to an impartial trial upon its own intrinsic merits, without taking into account the foolishness and ultraism of its especial advocates. Atropine, strychnia and morphia can, in many instances, be used hypodermically with the greatest possible advantage—with such favorable results as no other system of medication could possibly secure. This is the testimony not only of Wood, Hunter and Anstie, but of a large majority of the most intelligent members of the profession everywhere. Who has not witnessed the miraculous effects produced by the subcutaneous injections of morphia in affections especially characterized by nervous excitability? In a single moment it enters the blood, reaches the great nerve centres, and takes possession of them—establishing therein a veritable "imperium in imperio," beneath whose magic sway quiesence is inaugurated where the wildest disturbance reigned before. The subtle influence flashing over nature's electric wires to every portion of the system, not only whispers to each excited element "peace be still," but coerces the whole organism into a similar state of tranquility. Thus, as if by enchantment, the wrinkled brow grows calm again, the contracted muscles subside in repose, the recusant organs become submissive, and "nature's sweet restorer—balmy sleep," possesses the soul and fills it with dreams of Elyseum. Facts like these are too significant to be overlooked or disregarded. They speak in trumpet tones against the perverseness and fatuity of those who would discard so potent a remedy from the list of therapeutical agencies. Though innocent of the crime of *commission* in this regard, the enemies of hypodermic medication are guilty of *omissions* which are utterly inexcusable in these days of progress and enlightenment. If not *positively* derelict they are *negatively* so, to an extent that deserves condemnation and reprobation.

The indiscriminate employment of this remedy—the constant resort to hypodermic medication, under all circumstances—is, perhaps, more unphilosophical and improper than its entire abnegation. In many instances, even where pain and nervous excitability exist, it is positively and most peremptorily contraindicated, and the infatuation which induces sensible men to resort to this mode of treatment in such cases, is not simply a phase of empiricism, but an unequivocal mistake, amounting almost to criminality itself.

That extremes always meet in a common ground of error, is most signally and significantly illustrated in this instance —in an entire repudiation of an invaluable remedy by one class of physicians, and in such an employment of it by others as destroys its usefulness and renders it a source of detriment rather than of benefit to suffering humanity.

"*In medio tutissimus ibis!*"—Editorial in *Balto. Med. Bulletin.*

---

